# Revealing the Calcium Assisted Partial Catalytic Graphitization of Lignin-Derived Hard Carbon Anode and Its Electrochemical Behaviors in Sodium Ion Batteries

**DOI:** 10.3390/polym17040540

**Published:** 2025-02-19

**Authors:** Jungpil Kim, Sang-Hyun Lee, Junghoon Yang

**Affiliations:** 1 Carbon & Light Materials Group, Korea Institute of Industrial Technology, Jeonju 54853, Republic of Korea; jpkim@kitech.re.kr; 2 Lignum, Daejeon 34134, Republic of Korea; ceo@lignum.co.kr

**Keywords:** hard carbon, anode, lignin, catalytic graphitization, sodium-ion batteries

## Abstract

Among the various contenders for next-generation sodium-ion battery anodes, hard carbons stand out for their notable reversible capacity, extended cycle life, and cost-effectiveness. Their economic advantage can be further enhanced by using inexpensive precursors, such as biomass waste. Lignin, one of the most abundant natural biopolymers on Earth, which can be readily obtained from wood, possesses a three-dimensional amorphous polymeric structure, making it a suitable candidate for producing carbonaceous materials through appropriate carbonization processes for energy storage applications. In this work, we synthesized hard carbon using lignin containing CaSO_4_ to facilitate partial catalytic graphitization to improve the microstructural features, such as interlayer spacing, degree of disorder, and surface defects. Partial catalytic graphitization enables hard carbon to develop an ordered structure compared with hard carbon carbonized without CaSO_4_ as analyzed by X-ray diffraction, Raman spectroscopy, scanning/transmission electron microscopy, and X-ray photoelectron spectroscopy. The CaSO_4_-aided partially catalytic graphitized hard carbon (CCG-HC) exhibited improved electrochemical performance, showing a larger portion of the low-voltage plateau—an indicator typically associated with a highly ordered structure—compared to simply carbonized hard carbon (HC). Notably, CCG-HC delivered a reversible capacity of 237 mAh g^−1^, retained 95.6% of its capacity over 100 cycles at 50 mA g^−1^, and exhibited 127 mAh g^−1^ at 1.0 A g^−1^.

## 1. Introduction

The rapid expansion of renewable and clean energy technologies, such as solar and wind power, has spurred improvements in large-scale energy storage systems (ESS) to ensure their efficient utilization [1,2,3,4]. While lithium-ion batteries (LIBs) currently serve as the leading choice for rechargeable batteries in mobile electronics and electric vehicles due to their high energy density, long cycle life, and robust power density, their application in ESS is constrained by escalating costs, largely due to limited lithium reserves and uneven global distribution. Consequently, sodium-ion batteries (SIBs) have emerged as a promising next-generation alternative, owing to the low cost and abundant availability of sodium resources [5,6,7]. Furthermore, the similarity in working principles between SIBs and LIBs enhances their appeal. However, the distinct physicochemical properties of sodium ions compared to lithium ions necessitate the development of suitable electrode materials, including both cathodes and anodes, for SIBs. Unlike LIBs, which primarily utilize layered-structure materials, cathode materials for SIBs are being actively investigated to establish a wider range of options. These include layered oxides containing various transition metals [8,9,10,11,12], three-dimensional NASICON (Na-ion superionic conductor) compounds [13,14,15], and organic-based Prussian blue and Prussian white materials [16,17,18]. Meanwhile, on the anode side, high-capacity metal-based materials [19,20,21] and metal sulfide/oxide [22,23,24,25] are also receiving considerable attention.

One well-known manifestation of the differences in electrode materials between lithium ions and sodium ions is observed in graphite, the most commercialized anode material in LIBs [26]. Graphite exhibits inactivity for sodium ion storage due to the thermodynamic instability of forming binary graphite intercalation compounds (GICs) with sodium ions [27]. Therefore, extensive research is underway to identify alternative carbon anode materials more suitable for SIBs [28,29,30,31,32]. Among these materials, hard carbon—a non-graphitizable carbonaceous material—has attracted considerable attention due to its favorable interaction with sodium ions. By controlling parameters such as interlayer spacing, surface area, pore structure, and crystallinity, hard carbon can deliver high reversible capacity along with excellent cycling stability and rate capability [33,34,35,36,37].

The low-cost production of hard carbon from environmentally friendly biomass further enhances its appeal. Various biomass resources have been utilized as precursors for synthesizing hard carbon [38,39,40,41,42], and their composition can vary significantly, often including cellulose, hemicellulose, and lignin [43,44]. Among these, lignin—the most abundant natural biopolymer and a key structural component in most plants—exhibits particular promise for hard carbon synthesis, even when used alone [45]. Lignin is primarily generated as a by-product of the paper and pulp industries, and the expanding biofuel sector is anticipated to produce even larger quantities of lignin. However, lignin is largely discarded due to a lack of suitable applications [46,47]. Therefore, converting lignin into hard carbon as an anode material for SIBs could be a viable strategy [48,49]. In general, it is recognized that lignin, which consists of a three-dimensional amorphous polymeric structure, is primarily composed of three oxidatively coupled 4-hydroxyphenylpropanoid units that differ in their degree of methoxylation [50]. Despite its complex structural characteristics, lignin has relatively high carbon content (60–75%), enabling a high yield for hard carbon production. However, there remains a knowledge gap regarding how different physicochemical properties ultimately influence the structure of carbonaceous materials.

In this study, we investigated the relationship between the micro-structural properties and electrochemical behaviors of lignin-derived hard carbons as anodes for SIBs. In particular, we aimed to closely investigate how the microstructure of hard carbon affects the high-voltage sloping region and the low-voltage plateau region in the sodium ion storage process, which will be discussed later in this work. Lignin, a natural biopolymer, served as the precursor for synthesizing hard carbon. This study also examined how the presence or absence of CaSO_4_ during the carbonization of lignin impacts its microstructure. CaSO_4_ naturally forms in the lignin production process from biomass (e.g., wood), where H_2_SO_4_ is used and then neutralized with CaO. The presence of calcium can act as a catalyst during the carbonization process, promoting catalytic graphitization and thereby enhancing the crystallinity of hard carbon even at the same carbonization temperature [51,52]. We observed that the relative proportion of the ordered region increased if the hard carbon was synthesized in the presence of calcium species. Furthermore, these structural modifications were found to improve the electrochemical properties of hard carbon as anode materials for SIBs.

## 2. Materials and Methods

### 2.1. Materials Preparation

For the preparation of hard carbon anode materials for SIBs using lignin, CaSO_4_ containing lignin (CSL) provided by Lignum (Daejeon, Republic of Korea) was used as precursor without further purification. To study the structural changes during the carbonization process depending on the presence or absence of CaSO_4_ in lignin, CSL was carbonized under two conditions: one with CaSO_4_ (Ca-related components washed after carbonization; the CaSO_4_-aided partially catalytic graphitized hard carbon, CCG-HC) and the other without CaSO_4_ (CaSO_4_ washed before carbonization, HC). The carbonization process was carried out at 1000 °C for 2 h under an Ar atmosphere.

### 2.2. Material Characterization

The structural characteristics of the CSL-derived hard carbon samples were probed using powder X-ray diffraction (XRD, Ultima IV, Rigaku, Tokyo, Japan) and Raman spectroscopy (RAMANtouch, Nanophoton Corp., Osaka, Japan). The morphologies and microstructural properties of the lignin precursor and synthesized hard carbons were analyzed using a field-effect scanning electron microscope (FE-SEM, JSM-6700F, JEOL Ltd., Tokyo, Japan) and Cs-corrected transmission electron microscope (TEM, Titan G2 Cube 60-300, FEI, Hillsboro, OR, USA). Their chemical states were examined by X-ray photoelectron spectroscopy (Thermo/K-Alpha ESCA System, Thermo Fisher Scientific Inc., Waltham, MA, USA). The carbon content characteristics of materials were determined by thermogravimetric analysis (STA6000, Perkin Elmer, Waltham, MA, USA).

### 2.3. Electrochemical Analysis

The hard carbon electrode was fabricated by blending 80 wt% active materials (CCG-HC or HC), 10 wt% acetylene black, and 10 wt% polyvinylidene fluoride (PVdF) in an N-methylpyrrolidone (NMP) solvent. The resulting slurry was uniformly cast onto a copper foil using a doctor blade, followed by drying under vacuum at 100 °C for 5 h. Subsequent to drying, the electrodes were compressed and punched into circular shapes with a 1.4 cm diameter, achieving an average active material loading of 3.5 mg cm^−2^. Electrochemical measurements were conducted using CR 2032 coin-type cells assembled in an argon-filled glovebox, employing sodium metal foil as the counter electrode and 1 M NaPF_6_ in diethylene glycol dimethyl ether (DEGDME) as the electrolyte. The coin cells were subjected to galvanostatic charge–discharge tests at room temperature over a voltage range of 2.0–0.01 V (vs. Na/Na^+^) under various current densities, using a battery cycler (WBCS 3000, Wonatech, Seoul, Republic of Korea).

## 3. Results

**Figure 1:**
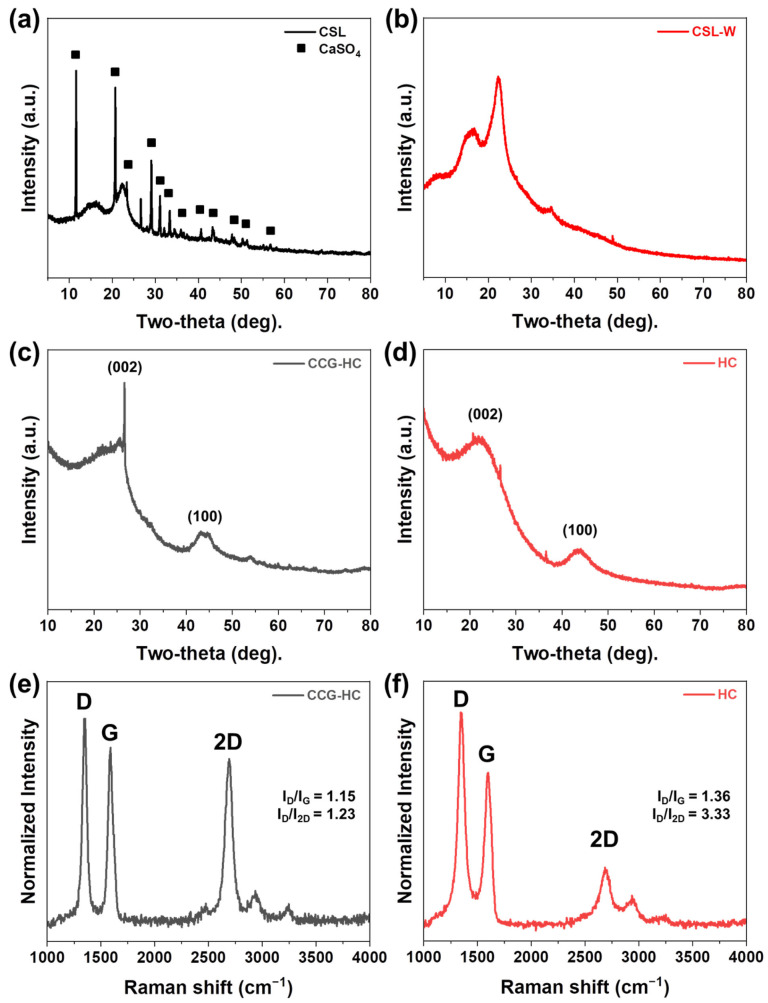
XRD pattern of (**a**) CSL, (**b**) CLS-W, (**c**) CCG-HC, and (**d**) HC. Raman spectra of (**e**) CCG-HC and (**f**) HC.

The structural and compositional properties of the lignin precursor and carbonized samples were analyzed by XRD analysis as shown in Figure 1. As-prepared lignin (CSL, CaSO_4_-containing lignin) appears to include CaSO_4_ formed through the reaction of CaO (used in the neutralization process) with sulfuric acid. The lignin phase is identified by relatively broad peaks around 15.8° and 20°, while CaSO_4_ is confirmed by its characteristic crystalline peaks (marked with squares) (Figure 1a). This CaSO_4_ can be easily removed through a washing step using distilled water, which is evidenced in Figure 1b. Here, the CaSO_4_ peaks disappear completely, leaving only the characteristic peaks of lignin. The washing process is abbreviated as “W”, and the CaSO_4_-free sample is referred to as CSL-W. In this study, we investigate the structural differences of hard carbon derived from the carbonization of lignin with and without CaSO_4_, as well as its electrochemical properties when used as an anode material for SIBs. Figure 1c,d present the XRD results of hard carbons prepared from lignin containing CaSO_4_ and lignin without CaSO_4_, respectively. The first sample, which was washed after heat-treating the CSL, is referred to as CCG-HC (calcium assisted partial catalytic graphitized hard carbon). In this sample, calcium-related impurities were removed by washing after the heat-treatment. As shown in Figure S1, CaSO_4_ was converted into CaS following carbonization together with lignin. Meanwhile, the second sample, which underwent a washing step before heat treatment, is designated simply as HC. Basically, when carbonized at 1000 °C in an inert atmosphere, two distinct diffraction peaks appear at approximately 24° and 43°, corresponding to the graphitic-like (002) and (100) planes, respectively. The broadness of these peaks indicates the formation of an amorphous carbon structure rather than a crystalline phase. However, while CCG-HC exhibits a narrow and sharp peak at around 26.6°, HC does not show such a strong peak, indicating microstructural differences between two samples. This suggests that the presence of CaSO_4_ influences the formation of the highly ordered hard carbon structure during the carbonization process by partial catalytic graphitization. In general, catalytic graphitization is a pathway to obtain more crystalline carbon at lower temperatures by using metals or metal compounds that temporarily dissolve and then re-precipitate carbon in a more ordered, graphitic arrangement [51,52]. Detailed structural differences between CCG-HC and HC in the local region were further analyzed using Raman spectroscopy, as shown in Figure 1e,f. The Raman spectra of carbonaceous materials identify three distinct bands: (1) the D band, arising from defects and/or disordered sp^3^ carbon configurations (~1355 cm^−1^); (2) the G band, indicative of sp^2^ carbon arrangements (~1585 cm^−1^); and (3) the 2D band, reflecting ordered structures similar to graphite (~2730 cm^−1^) [53,54,55,56,57]. For quantitative analysis, the Raman spectra of CCG-HC and HC were normalized. The intensity ratio between the D band and the G band (I_D_/I_G_) was calculated to quantitatively assess the crystallinity of the two different hard carbons. The I_D_/I_G_ ratios of CCG-HC and HC were ~1.15 and ~1.36, respectively. In addition, it was observed that CCG-HC exhibits a well-developed 2D band, which is attributed to the two-dimensionality of the carbon materials. The lower I_D_/I_G_ and I_D_/I_2D_ ratio of CCG-HC demonstrates that the presence of CaSO_4_ is effective in the formation of a highly ordered carbon structure, as well as being in good agreement with the XRD results.

**Figure 2:**
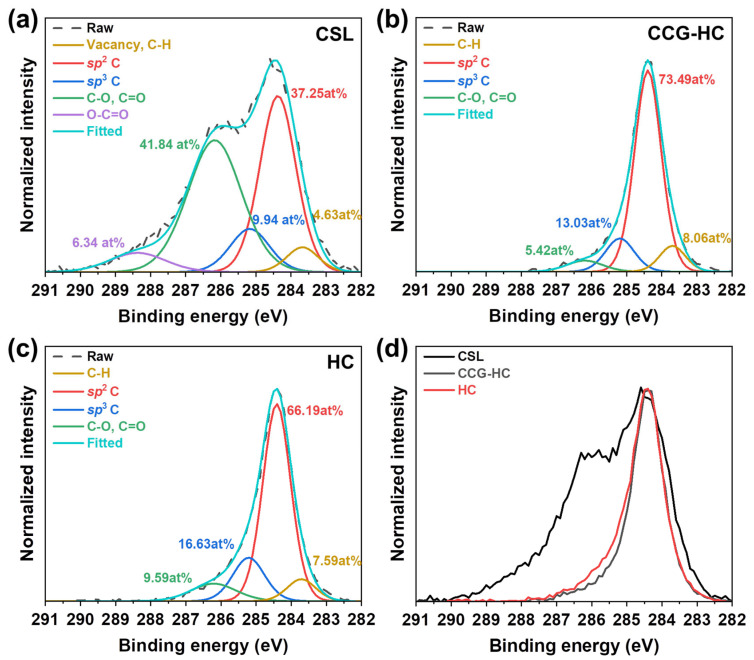
High resolution C 1s XPS spectra of CSL and CSL-derived hard carbons. Peak deconvolution of high resolution C1s spectra of (**a**) CSL, (**b**) CCG-HC, and (**c**) HC. (**d**) Comparison of normalized C1s spectra of all samples.

The chemical state of carbon atoms of CLS and CLS-derived hard carbons with respect to the presence of CaSO_4_ during the carbonization process was further investigated by XPS analysis, as shown in Figure S2 and Figure 2. Figure S2 presents the XPS analysis results for calcium and sulfur, confirming their removal from the samples via washing. In general, the C1s spectra of carbonaceous materials can be deconvoluted by several different species including vacancies/C-H (on edges), *sp*^2^ carbon, *sp*^3^ carbon, C-O/C=O, and O-C=O, which are located at 283.7 eV, 284.8 eV, 285.6 eV and 286.5 eV, and 289.2 eV, respectively [58,59]. The areal atomic ratios of each component of the samples are marked in the figure. As shown in Figure 2a, CSL exhibited a significant amount of oxygen functional groups, including C-O, C=O, and –O-C=O peaks, as expected given the molecular structure of lignin. As carbonization proceeded, most oxygen-containing functional groups disappeared, accompanied by the development of *sp*^2^ carbon and *sp*^3^ carbon peaks, as shown in Figure 2b,c. For CCG-HC, the *sp*^2^ C and *sp*^3^ C contents were 73.49 at% and 13.03 at%, respectively. In contrast, for HC, the *sp*^2^ C and *sp*^3^ C contents were 66.19 at% and 16.63 at%, respectively. As shown in Figure 2d, the full width at half maximum (FWHM) of the CCG-HC was narrower than that of HC due to a decreased amount of *sp*^3^ C and C-O/C=O components. The change in FWHM is an indicator demonstrating the effect of the partial catalytic graphitization on converting disordered carbon to ordered carbon with the help of CaSO_4_ during the carbonization process, as we already analyzed from the XRD and Raman analysis results. The analysis suggests that the presence of CaSO_4_ enhances the elimination of oxygen functional groups from the lignin precursor, thereby facilitating a more ordered arrangement of carbon atoms.

**Figure 3:**
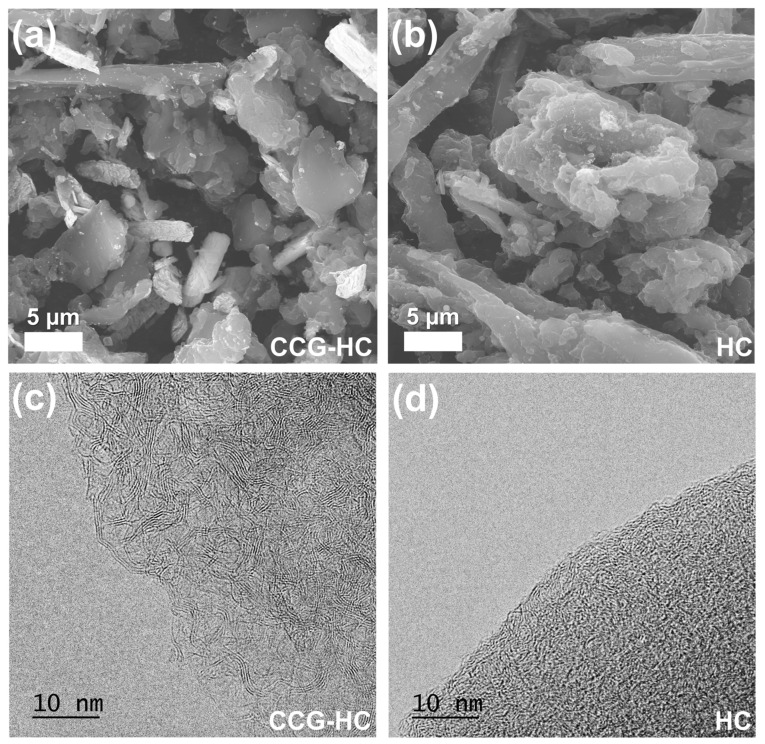
Electron microscopic analysis results of CCG-HC and HC. SEM image of (**a**) CCG-HC and (**b**) HC. TEM image of (**c**) CCG-HC and (**d**) HC, demonstrating differences in crystallinity between samples.

The morphological characteristics and microstructural characteristics of CCG-HC and HC were examined using microscopic analysis methods such as FE-SEM and cs-corrected TEM, as shown in Figure 3. The SEM images of CCG-HC and HC are shown in Figure 3a and Figure 3b, respectively. Both samples have an irregular particle morphology including round shape particles and short-rod shape particles, demonstrating that the synthesis process differences of partial catalytic graphitization for CCG-HC and simple carbonization for HC has no distinct effect on morphological changes. The round-shaped particles range in size from approximately 5 to 10 μm, while the short-rod-shaped particles have diameters of about 3 to 5 μm. The round-shaped particles originate from lignin, whereas the short-rod-shaped particles are fiber-like cellulose remaining after lignin extraction and purification. As shown in Figure S3, the morphological characteristics of both CCG-HC and HC closely resemble those of the lignin precursor; however, after partial catalytic graphitization and simple carbonization, the particles undergo noticeable shrinkage. Furthermore, the TGA analysis presented in Figure S4a shows that the lignin precursor undergoes a weight loss of up to 23 wt% when heated to 800 °C under a nitrogen atmosphere, suggesting that the yields of CCG-HC and HC derived from the lignin precursor may be similar. As shown in Figure S4b, the TGA results under an air atmosphere indicate residual masses of about 96.6 wt% for CCG-HC and 95.3 wt% for HC, revealing that CCG-HC has a slightly higher carbon content. This suggests that partial catalytic graphitization facilitates more efficient oxygen removal. To gain a deeper understanding of the carbon-atom arrangements in both CCG-HC and HC, high-resolution TEM analyses were carried out, as illustrated in Figure 3c,d. The CCG-HC sample exhibits a more ordered architecture, with distinct lattice fringes suggestive of stacked graphene layers. In contrast, HC features shorter and randomly oriented fringes, indicating significant defects and irregular atomic configurations that impede internal pore formation. These HR-TEM observations corroborate the findings from XRD and Raman spectroscopy, confirming that the presence of calcium species during the carbonization process promotes the emergence of localized ordered regions in hard carbon due to effect of partial catalytic graphitization.

**Figure 4:**
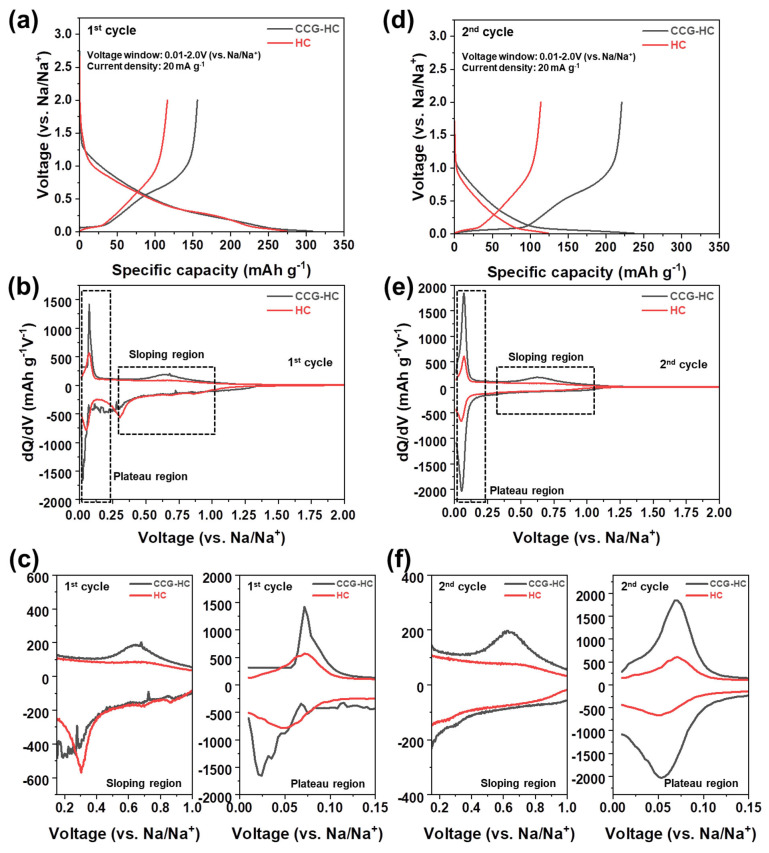
(**a**) Galvanostatic sodiation and desodiation profiles of CCG-HC and HC obtained at initial cycle and (**b**) corresponding dQ/dV plots. (**c**) A closer observation of the dQ/dV plots for the sloping region and plateau region at initial cycle. (**d**) Galvanostatic sodiation and desodiation profiles obtained for CCG-HC and HC at second cycle and (**e**) corresponding dq/dV plots. (**f**) A closer observation of the dQ/dV plots for the sloping region and plateau region at second cycle. (Voltage window: 0.01 V–2.0 V (vs. Na/Na^+^), current density: 20 mA g^−1^).

The electrochemical performance of CSL-derived hard carbons as anode materials for SIBs depending on the differences in microstructural properties according to the presence of CaSO_4_ during carbonization process were analyzed to study the relationship between structural properties and their electrochemical behaviors, as shown in Figure 4. The galvanostatic sodiation and desodiation profiles of CCG-HC and HC were evaluated in the voltage window of 0.01 V–2.0 V (vs. Na/Na^+^, hereafter) under current density condition of 20 mA g^−1^. In general, the electrochemical behaviors of hard carbon anodes can be classified by two main signals: the sloping region (above 0.1 V) and plateau region (below 0.1 V). Even though the mechanism of sodium storage in hard carbon has been controversial for many years, it is generally accepted that the high voltage sloping region is attributed to the adsorption of sodium ions on surface active sites including defects, turbostratic disorder sites, and/or chemical functionalities such as oxygen, while the low voltage plateau region is attributed to the pore-filling of sodium ions into the pore between micro-graphitic layers of hard carbons [60,61,62]. In the initial cycle, CCG-HC and HC exhibited a sodiation capacity of 308 mAh g^−1^ and 274 mAh g^−1^, respectively. In the following desodiation process at initial cycle, CCG-HC and HC exhibited a 155 mAh g^−1^ and 116 mAh g^−1^ desodiation capacity, respectively. It is noted that the both materials have a similar sodiation capacity, while the desodiation capacity for CCG-HC is much higher than that of HC in the initial cycle, indicating that structural characteristics clearly affect the electrochemical behaviors of the materials. The initial Coulombic efficiency (ICE) of CCG-HC and HC is 50.3% and 42%, respectively. Generally, the low ICE of carbon anode materials originates from unwanted electrolyte decomposition and corresponding solid-electrolyte interphase (SEI) formation in the initial cycle [63,64,65]. The detailed electrochemical behaviors were further studied by plotting the differential analysis (dQ/dV) plots, as shown in Figure 4b. For clearer comparison, the differential plots highlighting the sloping and plateau regions are presented in enlarged form in Figure 4c. As shown, the area of the differential plots from both the sloping and plateau region of CCG-HC is larger than that of HC, as expected by the capacity values observed in Figure 4a. However, the electrochemical behaviors seem similar, given the shapes of the galvanostatic sodiation and desodiation curves and dQ/dV plots. The galvanostatic sodiation and desodiation profiles and corresponding differential plots obtained at second cycle are shown in Figure 4d–f. As clearly seen in Figure 4d, the electrochemical behaviors of CCG-HC and HC became different compared with those obtained at initial cycle. In the second cycle, CCG-HC and HC exhibited a sodiation capacity of 237 mAh g^−1^ and 123 mAh g^−1^, respectively. The significantly reduced sodiation capacity at second cycle compared to the initial cycle is due to suppressed additional electrolyte decomposition by the already formed SEI layer, which has electronically insulating characteristics. Desodiation capacities of 221 mAh g^−1^ and 114 mAh g^−1^ were obtained for CCG-HC and HC, respectively. Based on the measured capacity, the CE of CCG-HC and HC at second cycle was 93.2% and 92.6%, respectively. Compared to HC, which shows a charge–discharge profile and capacity values in the second cycle similar to those in the initial cycle, CCG-HC displays changes in its sodiation and desodiation profiles and a significant increase in capacity values in the second cycle. As shown in Figure 4f, both CCG-HC and HC exhibit symmetrical dQ/dV curve shapes in the plateau region. However, in the sloping region, CCG-HC displays an asymmetric curve evidenced by a broad peak around 0.7 V during the desodiation process. This hysteresis in the voltage profiles can be attributed to differences in the energy barriers for ion diffusion during sodiation and desodiation in the more ordered structure of CCG-HC, manifesting as an overpotential. As noted earlier, this effect is driven by the expanded variety of sodium ion storage sites arising from the well-developed structure. These results further confirm that partial catalytic graphitization by calcium species imparts a more ordered structure to CCG-HC than to HC, in agreement with previous analyses. Therefore, the capacity increase in CCG-HC in the second cycle is attributed to the distinctly developed voltage plateau in the low voltage region.

**Figure 5:**
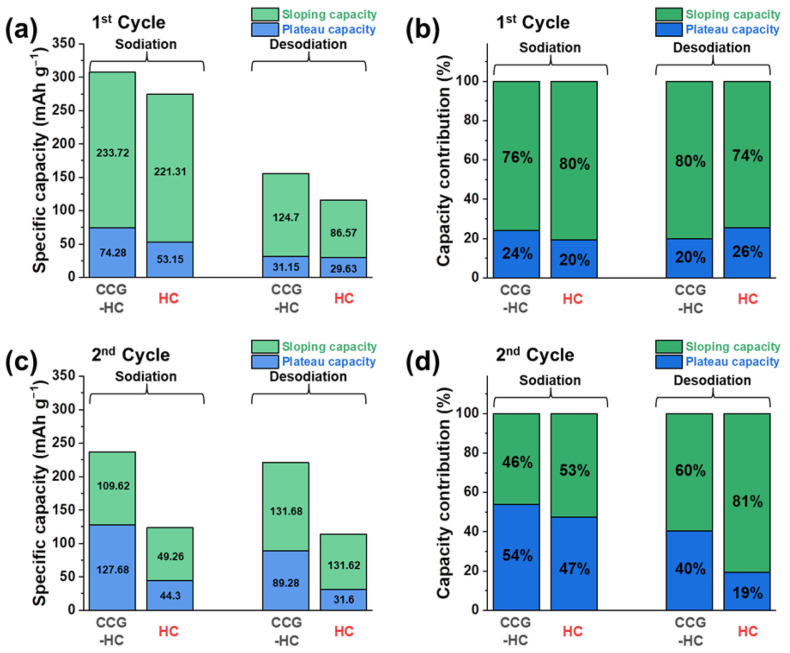
Analysis of the capacity contributions of CCG-HC and HC by voltage region. (**a**) Partitioning of the sloping and plateau capacities and (**b**) their respective contributions to total capacity during the initial cycle. (**c**) Partitioning of the sloping and plateau capacities and (**d**) their respective contributions to total capacity during the second cycle.

To gain a deeper understanding of how structural differences impact the electrochemical behavior of CCG-HC and HC, the capacity values were divided according to voltage ranges, as shown in Figure 5. In general, in the initial sodiation process, accompanied by unwanted redox reactions including electrolyte decomposition and SEI layer formation, the initial sodiation capacity is much higher than that of the desodiation capacity (Figure 5a). In Figure 5b, it can be confirmed that this unwanted reaction originates in the sloping region. Furthermore, Figure 5a,b indicate that the initial desodiation capacity is primarily generated in the sloping region at higher voltage. During the second sodiation process, CCG-HC exhibited a sloping capacity of 109.62 mAh g^−1^ and a plateau capacity of 127.68 mAh g^−1^, which account for 46% and 54% of the total sodiation capacity, respectively (Figure 5c). In comparison, HC exhibited a sloping capacity of 49.26 mAh g^−1^ and a plateau capacity of 44.3 mAh g^−1^, confirming its lower capacity relative to CCG-HC. From Figure 5d, it can be seen that CCG-HC not only exhibits a higher reversible capacity, but also demonstrates an increased proportion of plateau capacity at lower voltages, suggesting the potential for achieving higher energy density. During the desodiation process, a similar trend was also observed for CCG-HC and HC. These outcomes from the capacity contribution analysis align well with the expectations derived from the differential plot analysis. As mentioned, the low-voltage plateau region is attributed to insertion of sodium ions into micro-pores between graphene layers of hard carbon structure Therefore, it is reasonable that CCG-HC, which exhibits higher crystallinity due to partial graphitization from calcium species, benefits from the low-voltage region.

**Figure 6:**
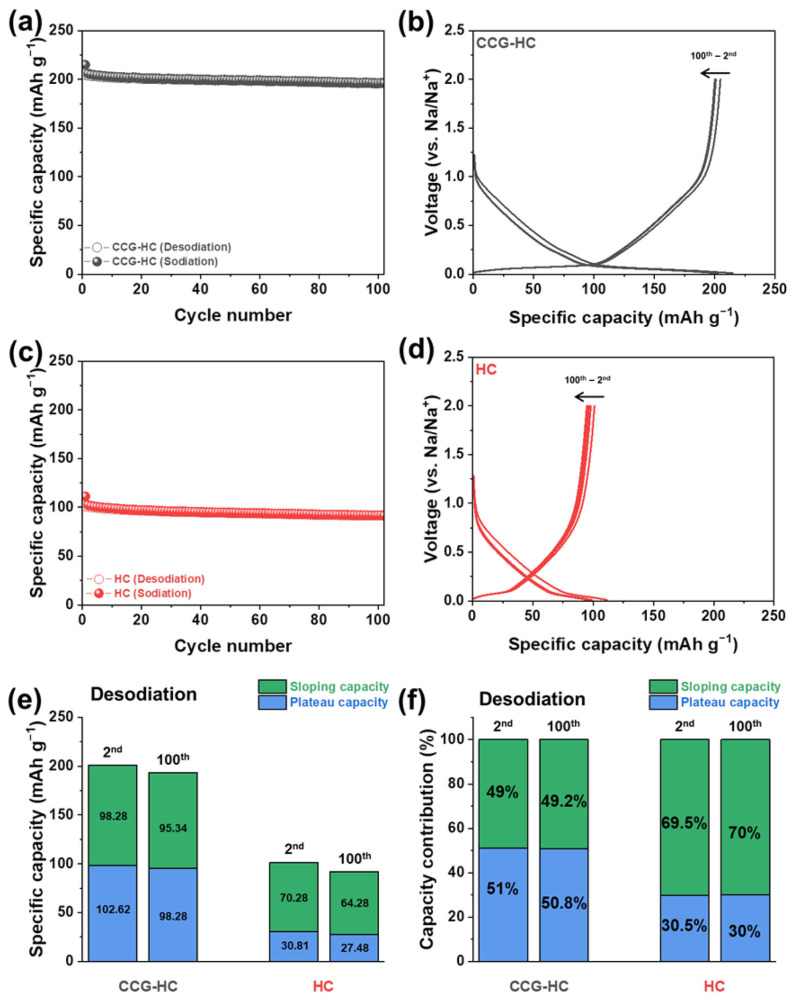
Cycling stability tests for CCG-HC and HC. (**a**) Cycle number vs. capacity plot and (**b**) corresponding voltage profiles of CCG-HC. (**c**) Cycle number vs. capacity plot and (**d**) corresponding voltage profiles of HC. (**e**) Partitioning of the sloping and plateau capacities and (**f**) their corresponding respective contributions to the total capacity of CCG-HC and HC, obtained from the 2nd and 100th cycles.

Figure 6 and Figure S5 presents the cycling stability test results of CCG-HC and HC up to 100 cycles at a current density of 50 mA g^−1^. As shown in Figure 6a, CCG-HC exhibits 195.7 mAh g^−1^ of desodiation capacity at the 100th cycle, and this value corresponds to 95.6% of the initial desodiation capacity. The voltage profiles obtained from cycling stability tests for CCG-HC are also plotted in Figure 6b. It is noticeable that the shapes of the sodiation and desodiation voltage profiles obtained at different cycle number are well overlapped due to the high reversibility of electrochemical processes toward repetitive sodiation and desodiation. Notably, it is clearly observed that the well-developed plateau region for CCG-HC is maintained during cycling stability tests. In the case of HC, it shows 91.4 mAh g^−1^ of desodiation capacity after 100 cycles, which represents a 90.4% capacity retention (Figure 6c). The voltage profiles of HC shown in Figure 6d show that the typical sloping shapes are maintained. To further clarify the changes in electrochemical processes of CCG-HC and HC, the specific capacity values depending on voltage region and their capacity contribution to total capacity between the 2nd and 100th cycle are compared in Figure 6e,f. In case of CCG-HC, the sloping capacity decreased from 98.28 mAh g^−1^ to 95.34 mAh g^−1^, and the plateau capacity decreased from 102.62 mAh g^−1^ to 98.28 mAh g^−1^. For HC, the sloping capacity decreased from 70.28 mAh g^−1^ to 64.28 mAh g^−1^, while the plateau capacity diminished from 30.81 mAh g^−1^ to 27.48 mAh g^−1^ after 100 cycles. From these results, it can be seen that, for CCG-HC, the capacity decreases evenly from both the sloping and plateau regions, whereas HC exhibits a greater capacity reduction in the sloping region. These findings suggest that the capacity loss in hard carbons is not tied to any particular voltage region, but is instead driven by structural differences.

**Figure 7:**
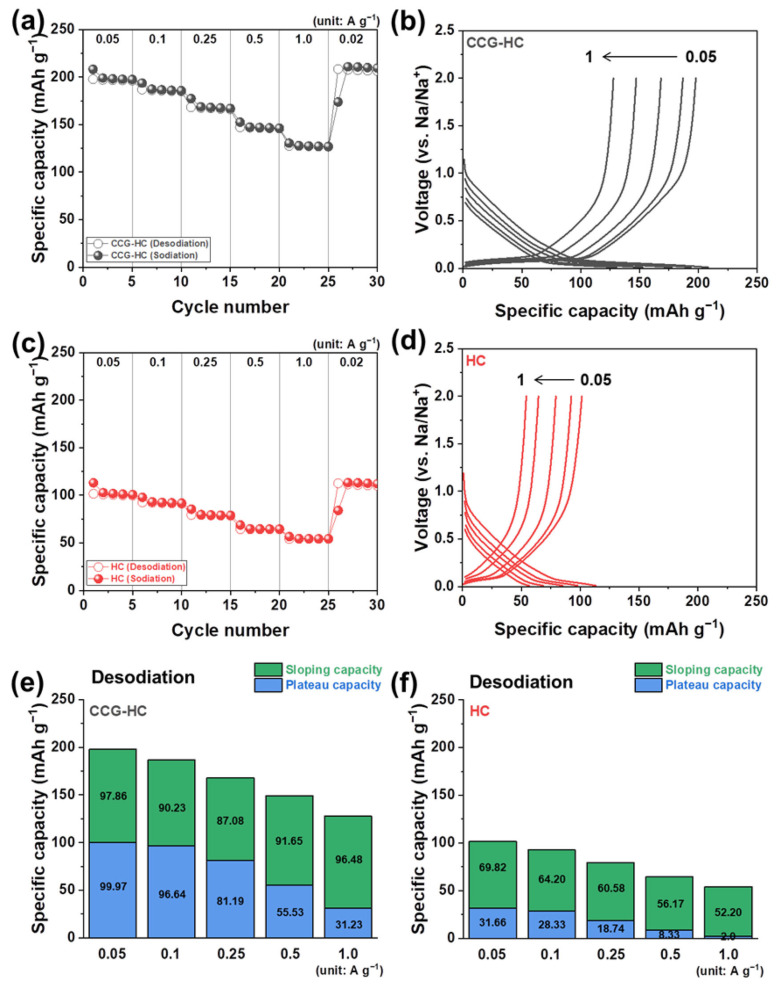
(**a**) Rate capability test results for CCG-HC obtained at current density conditions of 0.05, 0.1, 0.25, 0.5, and 1.0 A g^−1^ and (**b**) corresponding voltage profiles of CCG-HC. (**c**) Rate capability test results for HC obtained at current density conditions of 0.05, 0.1, 0.25, 0.5, and 1.0 A g^−1^ and (**d**) corresponding voltage profiles of HC. (**e**) Separation of sloping capacity and plateau capacity depending on current density of (**e**) CCG-HC and (**f**) HC, obtained from rate capability tests.

The effects of micro-structural differences between CCG-HC and HC on rate capability are analyzed in Figure 7 and Figures S6 and S7. The rate capability tests were conducted by changing the current density condition from 0.05 A g^−1^ to 1.0 A g^−1^. The desodiation capacity values of CCG-HC obtained at current densities of 0.05, 0.1, 0.25, 0.5, and 1.0 A g^−1^ were about 197, 186, 168, 147, and 127 mAh g^−1^, respectively (Figure 7a). The voltage profiles obtained from rate capability tests of CCG-HC are plotted in Figure 7b to clarify the electrochemical behaviors. Interestingly, under higher current density conditions, CCG-HC still exhibits a certain amount of capacity from the plateau region, indicating that the pore-filling sodium ion storage behaviors are still effective. In the case of HC, desodiation capacities of about 101, 92, 79, 64, and 54 mAh g^−1^ were obtained under current density conditions of 0.05, 0.1, 0.25, 0.5, and 1.0 A g^−1^, respectively (Figure 7c). The galvanostatic sodiation and desodiation profiles of HC are also plotted in Figure 7d. As clearly observed, CCG-HC exhibits higher capacity values compared to HC under all current density conditions. The capacity values of CCG-HC and HC, separated by voltage regions according to current density, along with their contributions to the total capacity, are presented in Figure 7e,d and Figure S7. As shown in Figure 7e, under relatively lower current density conditions of 0.05 A g^−1^ and 0.1 A g^−1^, CCG-HC exhibits a higher plateau capacity than sloping capacity. However, as the current density increases, the sloping capacity surpasses the plateau capacity. These results indicate that the pore-filling sodium ion storage behavior is kinetically hindered moreso than the sodium ion adsorption behavior. In the case of HC, at the lowest current density of 0.05 A g^−1^, the sloping capacity is higher than the plateau capacity. As the current density increases, the plateau capacity decreases sharply, and under a current density of 1.0 A g^−1^, most of the capacity originates from the sloping region. These findings can be attributed to the kinetically favored sloping region compared with the plateau region under higher current density conditions. Under low current density conditions, the slower reaction rate allows sodium ions sufficient time to insert into and extract from the ordered carbon structure. However, different and complex environments for sodium ion insertion and extraction occur if the current density increases. As the electrochemical reaction takes place over increasingly shorter durations, only the disordered region, where sodium ions are stored (corresponding to the sloping region), remains accessible. Consequently, the ordered region becomes more and more restricted in the electrochemical reaction. Nonetheless, the fact that CCG-HC exhibits a higher capacity than HC under all current density conditions suggests that it is crucial to appropriately control the microstructure of hard carbon so that disordered and ordered regions are harmoniously formed within the structure.

## 4. Conclusions

In summary, we synthesized hard carbon anode materials using the natural polymer lignin through a controlled carbonization process. These lignin-derived hard carbons were prepared under varying conditions—specifically in the presence or absence of CaSO_4_, a byproduct of lignin production—to evaluate the influence of CaSO_4_ through partial catalytic graphitization on the resulting material’s structure and performance. It was confirmed that the microstructural properties of hard carbons became ordered if the material was carbonized in the presence of CaSO_4_ as a result of partial catalytic graphitization under the same carbonization temperature. We investigated the correlation between the microstructural properties of lignin-derived hard carbons and their electrochemical performance as anode materials for SIBs, aiming to gain insights into how microstructure influences sodium ion storage behaviors. Our investigations revealed that hard carbon anodes exhibit two primary electrochemical behaviors: (1) A sloping region corresponding to sodium ion adsorption at surface active sites, such as defects, turbostratic disorder, and heteroatoms, and (2) a plateau region arising from sodium ion filling into micro-pores between well-ordered micro-graphitic layers. As the presence of calcium ions promoted partial catalytic graphitization and thus formed hard carbon with a highly ordered structure, the formation of the plateau region and an increase in capacity was observed in CCG-HC. As a result, CCG-HC carbonized with calcium ions exhibited a reversible capacity of 237 mAh g^−1^ by obtaining the values from both the sloping and plateau regions at the same time. During cycling stability tests, CCG-HC showed a capacity value of 195.7 mAh g^−1^ with retention of 95.6% up to 100 cycles when measured at a current density of 50 mA g^−1^. In terms of rate capability, CCG-HC exhibited a reversible capacity of 127 mAh g^−1^ at a current density of 1.0 A g^−1^. The improved electrochemical performance of CCG-HC compared to HC can be attributed to the formation of an ordered structure through partial catalytic graphitization by calcium ions, as evidenced by the development of a plateau region in the electrochemical processes. These results highlight the importance of properly controlling the microstructure to improve the performance of hard carbon anodes, and demonstrate that the partial catalytic graphitization method proposed in this study can generate ordered structures even at lower temperatures.

## Data Availability

Raw data are available upon request from corresponding author.

## Supplementary Materials

The following supporting information can be downloaded at: https://www.mdpi.com/article/10.3390/polym17040540/s1, Figure S1: XRD pattern of CSL-HC; Figure S2: XPS Ca2p and S2p spectra of CSL, CSL-BW, and CCG-HC-BW. Figure S3: SEM image of lignin precursor for CCG-HC and HC. Figure S4 TGA curves of lignin precursor, CCG-HC, and HC. Figure S5: Comparison of cycling stability of CCG-HC and HC. Figure S6: Comparison of rate capability of CCG-HC and HC. Figure S7: Capacity contribution under different current density obtained from rate capability test.
