# Supplementary material for: Revealing the Calcium Assisted Partial Catalytic Graphitization of Lignin-Derived Hard Carbon Anode and Its Electrochemical Behaviors in Sodium Ion Batteries
Source: Polymers (Basel). 2025 Feb 19;17(4):540. doi: 10.3390/polym17040540 (PMC11859861; doi:10.3390/polym17040540)
Supplement: Supplementary file 1 [file polymers-17-00540-s001.zip › polymers-3457948-supplementary.pdf]

Supplementary file

Revealing the Calcium Assisted Partial Catalytic Graphitization of Lignin-Derived Hard Carbon Anode and Its Electrochemical Behaviors in Sodium Ion Batteries

Jungpil Kim [1], Sang-Hyun Lee [2] and Junghoon Yang [1,*]

[1] Carbon & Light Materials Group, Korea Institute of Industrial Technology, Jeonju 54853, Republic of Korea; jpkim@kitech.re.kr
[2] Lignum, Daejeon 34134, Republic of Korea; ceo@lignum.co.kr
* Correspondence: jyang@kitech.re.kr

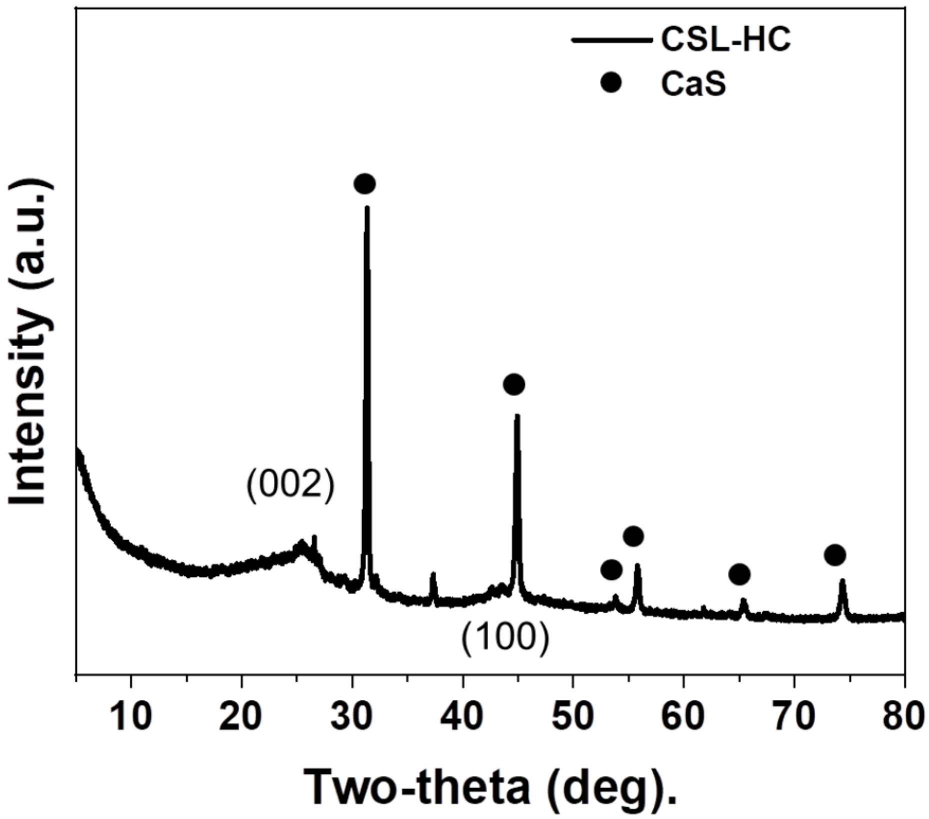

Figure S1. XRD pattern of CSL-HC.

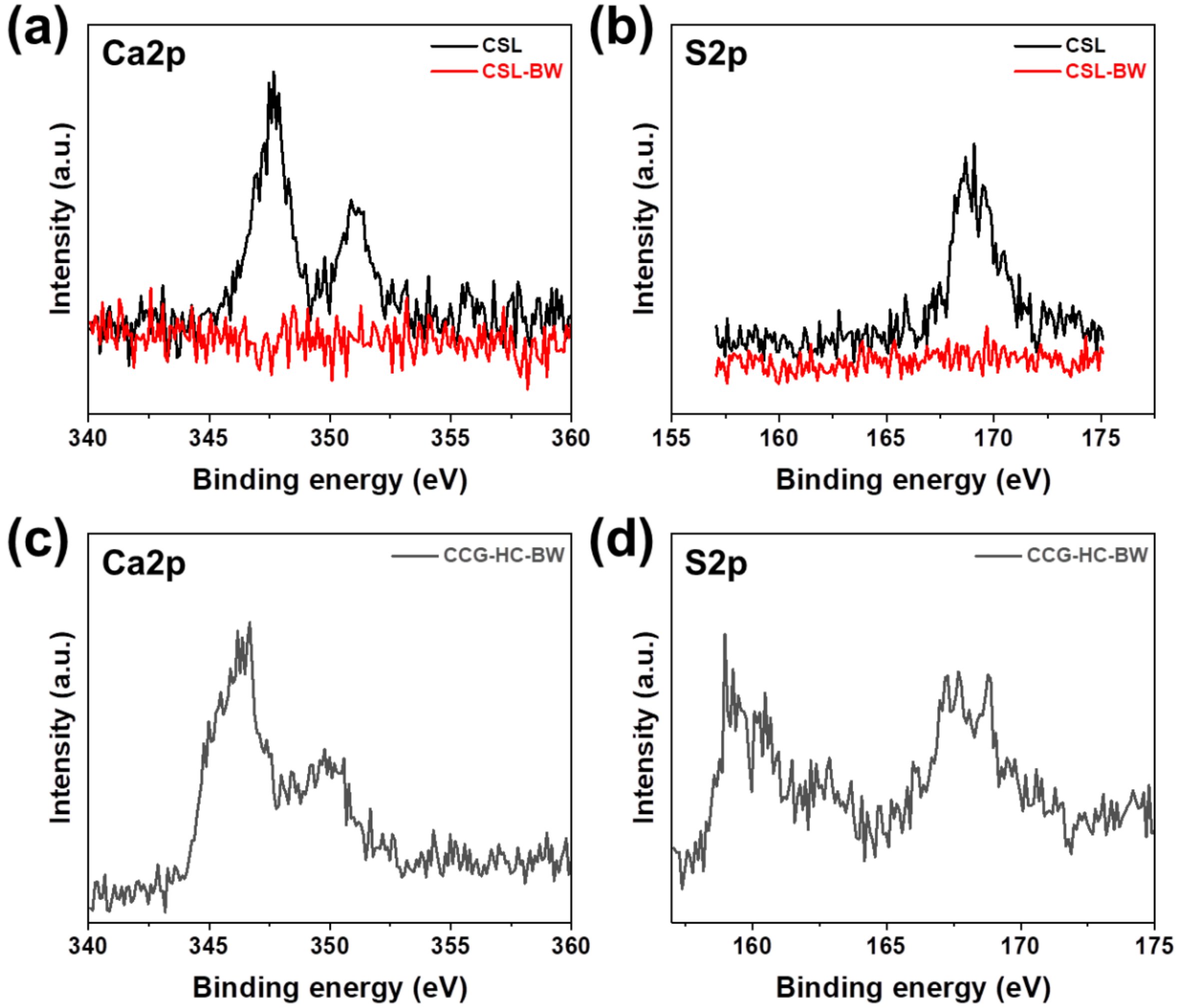

Figure S2. XPS (a) Ca2p and (b) S2p spectra of CSL, CSL-BW. (c) XPS Ca2p and (d) S2p spectra of CCG-HC-BW.

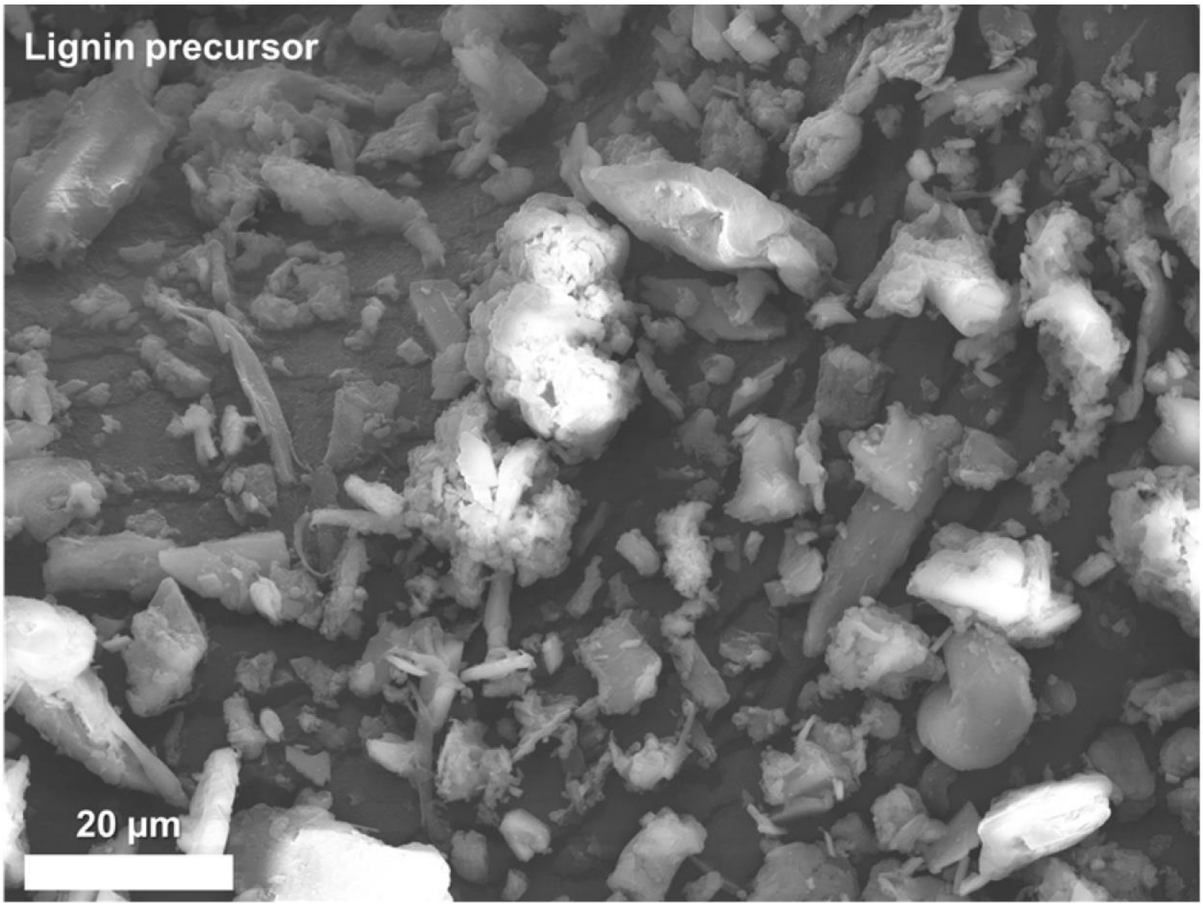

Figure S3. SEM image of lignin precursor for CCG-HC and HC.

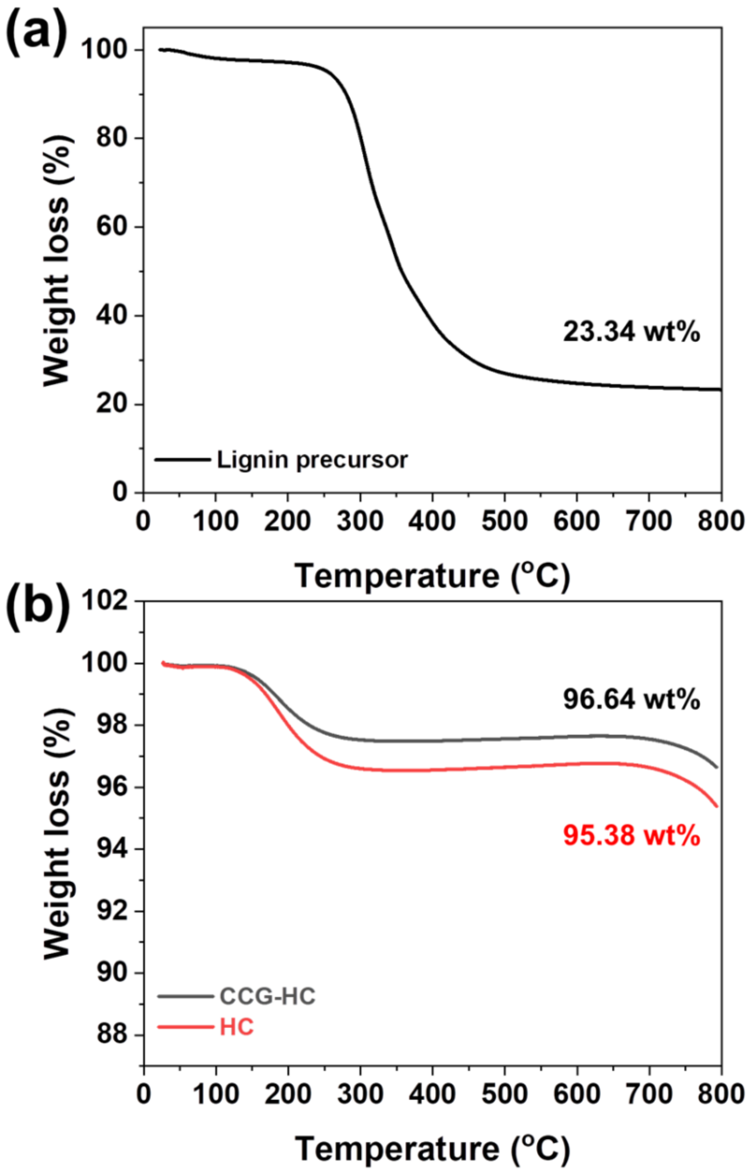

Figure S4. TGA curves of (a) lignin precursor in nitrogen atmosphere and (b) CCG-HC and HC in air atmosphere.

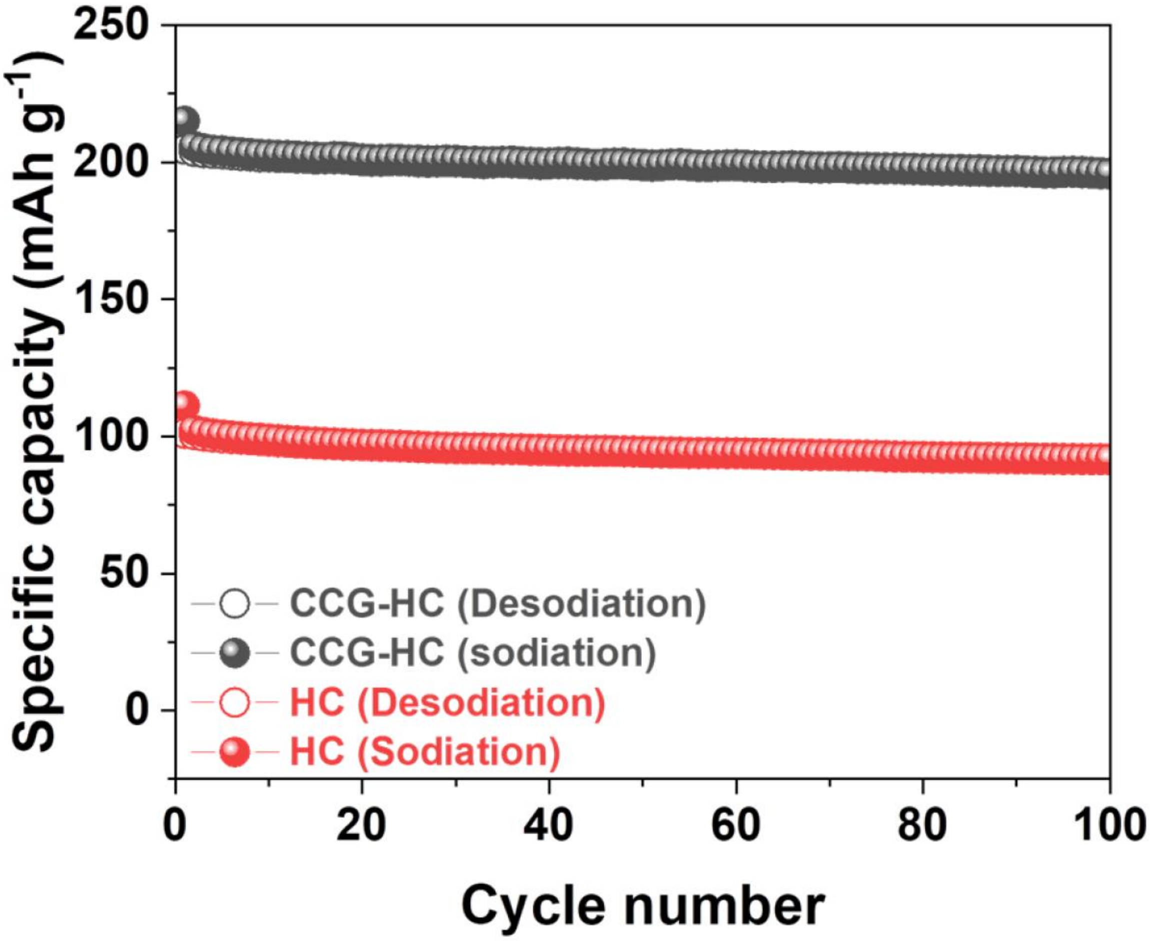

Figure S5. Comparison of cycling stability of CCG-HC and HC.

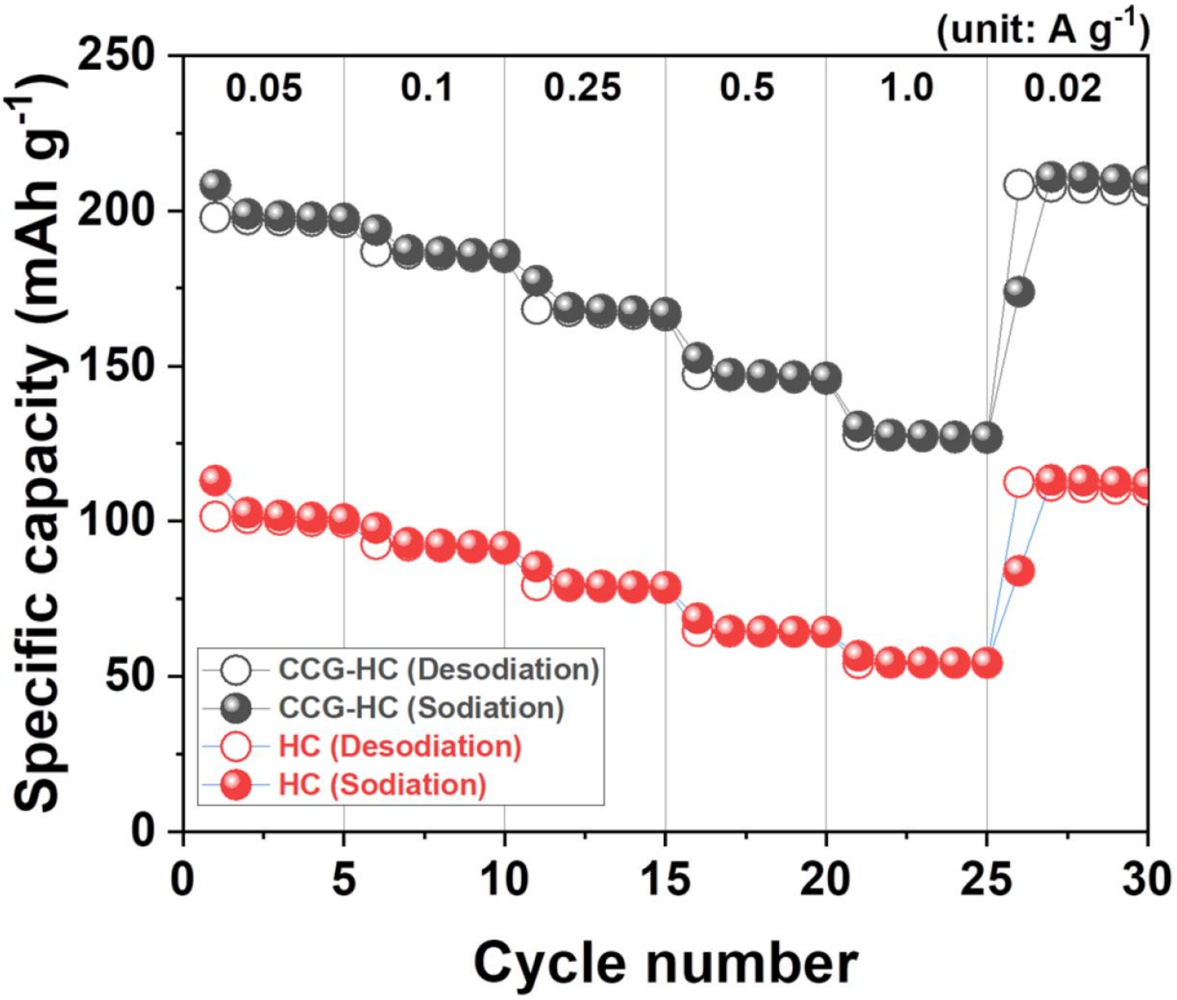

Figure S6. Comparison of rate capability of CCG-HC and HC.

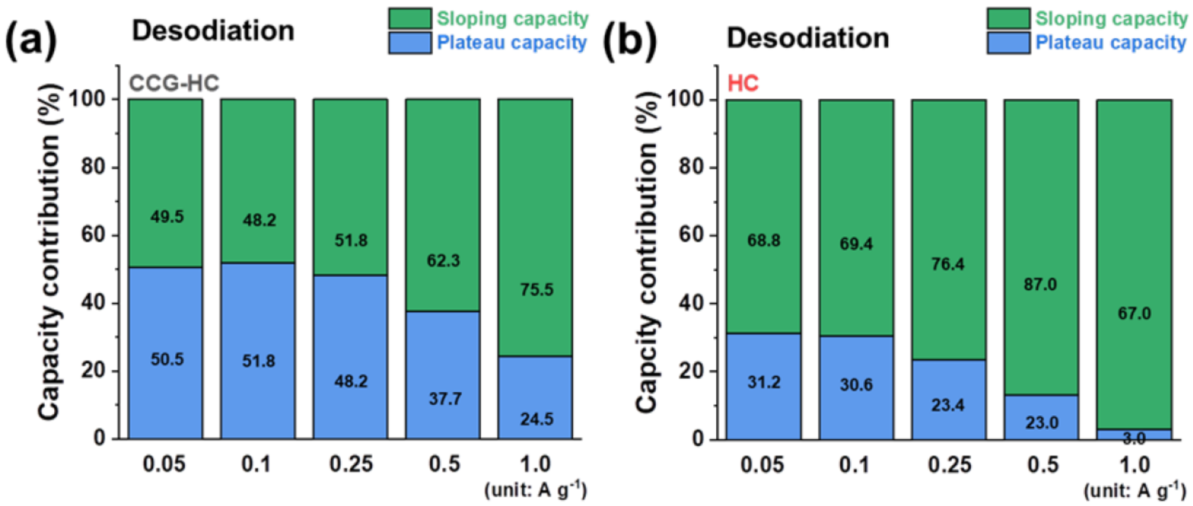

Figure S7. Capacity contribution of (a) CCG-HC and (b) HC under different current density obtained from rate capability tests.
